# *penner/lgl2* is required for the integrity of the photoreceptor layer in the zebrafish retina

**DOI:** 10.1242/bio.041830

**Published:** 2019-04-15

**Authors:** Satu Kujawski, Mahendra Sonawane, Elisabeth Knust

**Affiliations:** 1 Max-Planck-Institute of Molecular Cell Biology and Genetics, Pfotenhauerstrasse 108 01307 Dresden, Germany; 2 Tata Institute of Fundamental Research, Department of Biological Sciences, Homi Bhabha Road, Navy Nagar, Colaba, Mumbai 400005, India

**Keywords:** Apico-basal polarity, Adhesion, Plexiform layer, Lamination

## Abstract

The vertebrate retina is a complex tissue built from multiple neuronal cell types, which develop from a pseudostratified neuroepithelium. These cells are arranged into a highly organized and stereotypic pattern formed by nuclear and plexiform layers. The process of lamination as well as the maturation and differentiation of photoreceptor cells rely on the establishment and maintenance of apico-basal cell polarity and formation of adhesive junctions. Defects in any of these processes can result in impaired vision and are causally related to a variety of human diseases leading to blindness. While the importance of apical polarity regulators in retinal stratification and disease is well established, little is known about the function of basal regulators in retinal development. Here, we analyzed the role of Lgl2, a basolateral polarity factor, in the zebrafish retina. Lgl2 is upregulated in photoreceptor cells and in the retinal pigment epithelium by 72 h post fertilization. In both cell types, Lgl2 is localized basolaterally. Loss of zygotic Lgl2 does not interfere with retinal lamination or photoreceptor cell polarity or maturation. However, knockdown of both maternal and zygotic Lgl2 leads to impaired cell adhesion. As a consequence, severe layering defects occur in the distal retina, manifested by a breakdown of the outer plexiform layer and the outer limiting membrane. These results define zebrafish Lgl2 as an important regulator of retinal lamination, which, given the high degree of evolutionary conservation, may be preserved in other vertebrates, including human.

## Abstract (precis)

**Summary:** Knockdown of *penner/lgl2* leads to a breakdown of the outer plexiform layer and the outer limiting membrane in the zebrafish retina due to impaired cell adhesion.

## INTRODUCTION

Tissue formation is a complex process that requires a plethora of regulatory mechanisms in order to build a defined structure capable of executing tissue-specific functions. The vertebrate retina is an ideal model and has been used extensively to study the basic principles involved in tissue formation, including cell fate specification, differentiation of neuronal cell types, cell-cell and cell-matrix adhesion and pattern formation (Amini et al., 2017; Hoon et al., 2014; Stenkamp, 2015). The mature retina consists of one glial and five neuronal cell types, which are organized into five distinct layers. Three layers are formed by the cell bodies: the outer nuclear layer (ONL), comprising the cell bodies of the photoreceptor cells (PRCs), the inner nuclear layer (INL), containing cell bodies of horizontal, bipolar, amacrine and Müller glia (MG) cells, and the ganglion cell layer (GCL), whose neurons send their axons into the brain. These layers are separated by the outer and inner plexiform layers (OPL and IPL), which encompass the synapses between the different cell types. The highly stereotypic organization of these layers is a prerequisite to build the correct connections between the different cells of the retina, which in turn ensure the formation of functional neuronal networks required to transmit the light-induced signal from the PRCs into the visual cortex. Any defect that impairs cell fate specification or neuronal layering can result in impaired retinal function and loss of vision.

During retinal development, all neurons, including PRCs, are generated from a polarized, pseudostratified neuroepithelium. Similar to other epithelia, proper development of the retinal neuroepithelium depends on apico-basal cell polarity and on specialized cell-cell and cell-matrix junctions, which warrant tissue integrity. Apico-basal polarity in most epithelia is established and maintained by the evolutionarily conserved apical Crumbs (Crb) and Par3-Par6-aPKC complexes and by the basolateral Scribble (Scrib)-Discs large (Dlg)-Lethal(2) giant larvae (Lgl) and the Yurt-Coracle modules (Bazellieres et al., 2009; Bulgakova and Knust, 2009; Flores-Benitez and Knust, 2016; Goldstein and Macara, 2007; Laprise et al., 2009; Rodriguez-Boulan and Macara, 2014). These complexes are required to subdivide the plasma membrane into an apical and a basolateral domain and are required to position the apical junctions, such as adherens junctions and tight junctions. The apical plasma membrane is often highly differentiated to fulfil cell-type specific functions. In vertebrate PRCs, for example, the apical membrane strongly expands to form the outer segment (OS), which harbors the light-sensitive cone and rod visual pigments. Loss or overexpression of individual polarity proteins can affect the proper establishment and/or maintenance of apico-basal cell polarity and often results in impaired adhesion and breakdown of tissue integrity (Bilder, 2004; Bilder et al., 2000, 2003; Chalmers et al., 2005; Tanentzapf and Tepass, 2003; Yamanaka et al., 2003). These phenotypes underscore the importance of polarity genes and their proper regulation for the development and function of epithelial tissues.

In the vertebrate eye, the function of the apical polarity complexes has been associated with correct retinal lamination and PRC maturation. Loss of mouse *Crb1*, for example, induces defects in cell polarization and adhesion. This is manifested by the disruption of the outer limiting membrane (OLM), an adhesion zone between the PRCs, and the formation of rosettes (van de Pavert et al., 2004). Similarly, conditional knockdown of the mouse gene encoding Pals1 (protein associated with tight junctions 1)/Mpp5 (membrane-associated palmitoylated protein 5), a direct binding partner of Crb proteins, results in impaired differentiation of PRCs and of the OPL and in defects in retinal layering (Park et al., 2011) [reviewed in (Alves et al., 2014)]. Loss of function of zebrafish *crb2a* (*oko meduzy*, *ome*) impairs the stratified retinal organization as a result of defective adhesive interactions (Malicki and Driever, 1999), while overexpression of Crb2a impacts on the size of rod inner and OSs (Hsu and Jensen, 2010). In human, loss of *CRB1* or *CRB2* results in retinitis pigmentosa, one of the most severe retinal dystrophies leading to blindness (Chen et al., 2018; den Hollander et al., 1999) [reviewed in (Bujakowska et al., 2012; Slavotinek, 2016)].

In contrast to the apical polarity complex, the role of the components of the basal complexes in regulating retinal morphogenesis or photoreceptor polarity in vertebrates is less well understood. Dlg1, Scrib and Lgl1, originally identified in *Drosophila* as tumor suppressor genes (Bilder, 2004; Bilder et al., 2000; Gateff, 1978), are widely expressed in the adult mouse retina, including the GCL, INL, OPL, ONL and the retinal pigment epithelium (RPE) (Vieira et al., 2008). In the developing retina, Dlg1 and Scrib are both expressed in the OPL, OLM and in the RPE (Nguyen et al., 2005). However, their function in retinal development has not been studied so far.

Here, we set out to study the role of one of the two orthologs of *lgl*, *penner* (*pen*)/*lgl2*, in the zebrafish retina. The zebrafish is ideally suited to study retinal development due to its *ex utero* development and the transparency of the embryos. Many mutations affecting the development and function of the zebrafish retina have been identified in forward and reverse genetic screens (Karlstrom et al., 1996; Malicki et al., 1996; Trowe et al., 1996). Since human daytime vision largely relies on cone PRCs, the cone-dominated retina of the zebrafish provides a suitable tissue to study retinal development and vision. This has established the zebrafish retina as an excellent vertebrate model to unravel the genetic and molecular basis of human eye diseases (Bibliowicz et al., 2011; Blanco-Sánchez et al., 2017; Fadool and Dowling, 2008; Hoon et al., 2014; Stenkamp, 2015). So far, only *lgl1* function has been studied during early retinal development of the zebrafish. Retinal neuroepithelial cells with reduced Lgl1 levels maintain overall polarity and junctions, but have an enlarged apical plasma membrane domain, resulting in increased Notch signaling activity and reduced rates of neurogenesis (Clark et al., 2012).

The role of *pen/lgl2* in retinal development, however, has not been investigated so far, and its functions in later stages of PRC differentiation or maintenance are unknown. Animals mutant for *pen/lgl2* die around 6 days post fertilization (dpf), exhibiting an epidermal overgrowth phenotype and lack of hemidesmosomes in the basal layer of the larval epidermis (Sonawane et al., 2005). Furthermore, the basal epidermal cells exhibit a reduction in E-cadherin localization, undergo epithelial-mesenchymal transition (EMT) and migrate to ectopic locations due to the activation of EGF-receptor (ErbB) signaling (Reischauer et al., 2009). In addition, loss of *pen/lgl2* results in abnormal basolateral transport of E-cadherin in Kupffer's vesicle (KV), a ciliated epithelium essential for left-right asymmetry of the embryo. As a consequence, adhesion is affected, and cells exhibit reduction in cilia number and length (Tay et al., 2013). These results underscore the role for zebrafish Lgl2 in the control of polarized trafficking, apicobasal compartmentalization and cellular adhesion.

Here, we analyzed the role of *pen/lgl2* in the zebrafish retina. We show that Lgl2 is expressed in the developing retina during larval and juvenile stages. Yet, in *pen/lgl2* homozygous mutant larvae, lamination of the retina is not affected, and PRCs differentiate normally. Also, *pen/lgl2* mutant blastomeres transplanted to a wild-type retina differentiate into PRCs and survive to juvenile stages. However, additional knockdown of the maternal component leads to a breakdown of PRC layer integrity and disorganization of the distal retina, demonstrating the importance of Lgl2 for the development of an intact PRC layer.

## RESULTS

### Lgl2 is localized basolaterally in zebrafish photoreceptors and RPE cells

**Fig. 1.**
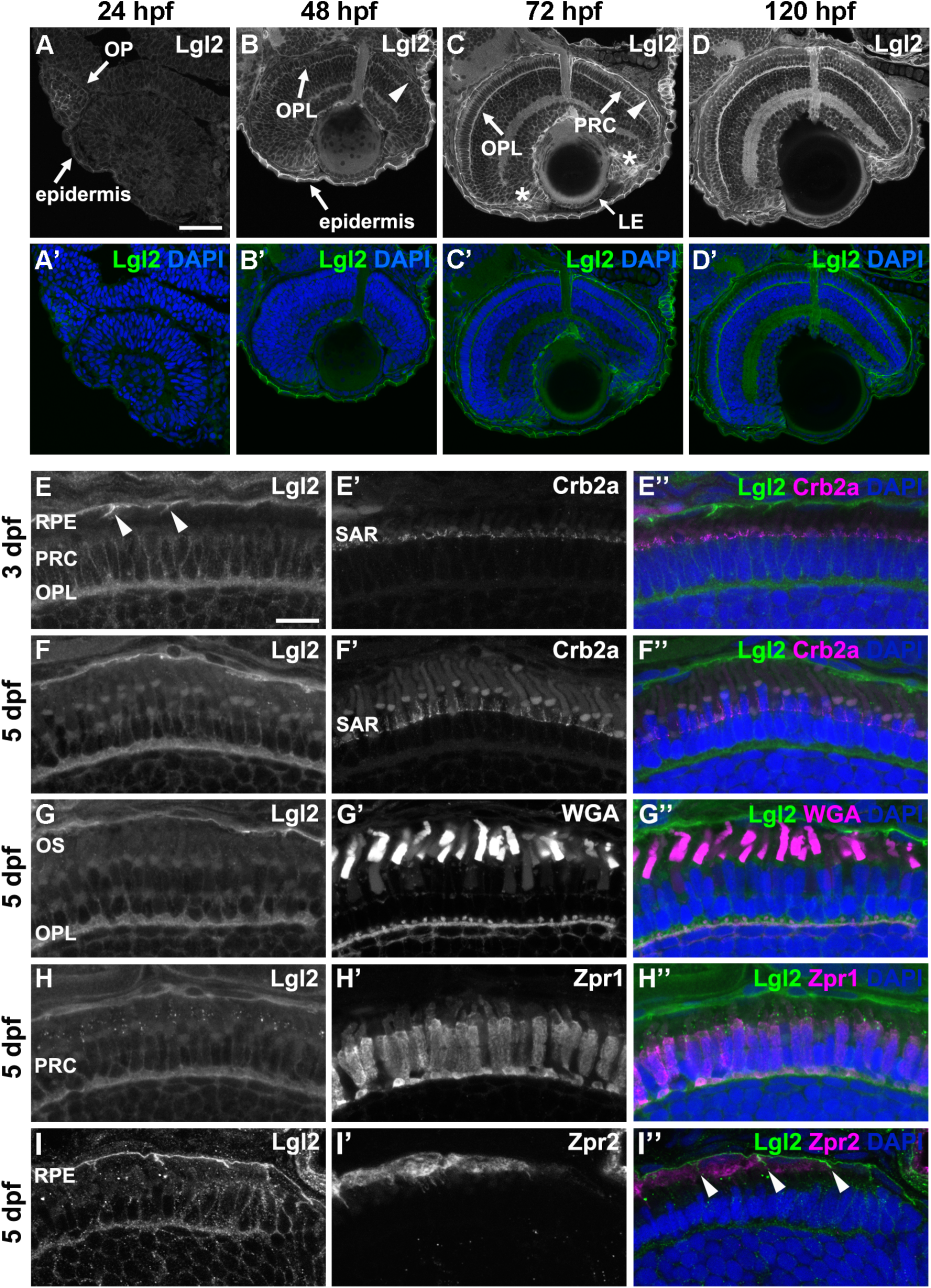
**Lgl2 expression is upregulated in the retina by 72** **dpf and localizes basolaterally in photoreceptor and RPE cells.** Immunostaining of transverse retinal sections. (A–D′) Lgl2 expression during retinal development: A,A′, 24 hpf; B,B′, 48 hpf; C,C′, 72 hpf; D,D′, 120 hpf. Lgl2 expression is upregulated by 72 hpf in the RPE (C, arrowhead) and the OPL. Asterisks in C denote expression in the ciliary marginal zone (CMZ). OP, olfactory placode; OPL, outer plexiform layer; PRC, photoreceptor cell layer; LE, lens epithelium. Scale bar: 50 µm. (E–E″) At 3 dpf, Lgl2 (E) localizes basolaterally in the PRC and in the RPE. Lgl2 staining does not overlap with that of Crb2a (E′), which localizes to the subapical region (SAR). Arrowheads in E denote lateral Lgl2 localization in RPE cells. E″ shows merged image. (F–I″) Lgl2 localization at 5 dpf. (F–F″) Co-staining of Lgl2 with Crb2a shows that Lgl2 staining remains basolateral as PRCs mature. (G–H″) Co-staining of Lgl2 (G,H) with WGA (G′) or Zpr1 (H′) illustrates Lgl2 localization in the OPL. (I–I″) Co-staining of Lgl2 (I) with Zpr2 (I′) reveals basolateral expression in the RPE (arrowheads in I″ denote lateral localization). OS, outer segments. Scale bar: 10 µm.

The tumor suppressor protein Lgl is localized at the basolateral membrane of many epithelial cells of different species (Cao et al., 2015; Grifoni et al., 2013). This also applies for Lgl2, one of the two paralogs in zebrafish, which is restricted to the basolateral cell cortex of larval outer epidermal, or peridermal, cells (Sonawane et al., 2009). Given the highly polarized nature of PRCs and the single-layered RPE, we set out to study the localization and function of Lgl2 in the zebrafish retina. Lgl2 protein levels in the retinal pseudostratified neuroepithelium at 24 h post fertilization (hpf) are low in comparison to its expression level in the epidermis and the olfactory patch (Fig. 1A, arrows). At 48 hpf, when the different retinal layers are forming, overall faint Lgl2 expression is detected in the retina (Fig. 1B). Expression in the OPL and the RPE becomes more pronounced by 72 hpf, when the expression level in the RPE is comparable to that in the epidermis (Fig. 1C, arrowhead). Lower expression of Lgl2 is observed in the PRC layer (Fig. 1C, arrow), the ciliary marginal zone (CMZ, Fig. 1C, asterisks) and the lens epithelium (LE, Fig. 1C, arrow). The same expression pattern is detected at 5 dpf (Fig. 1D).

To characterize Lgl2 localization in more detail in the zebrafish RPE and PRCs, we co-stained retinal sections with anti-Lgl2 in combination with known retinal markers (Fig. 1E–I″). Lgl2 is restricted to the basolateral membrane at 3 dpf (Fig. 1E) and 5 dpf (Fig. 1F) and does not show overlap with Crb2a (Fig. 1E′–F″), which marks the subapical region (SAR) of PRCs (Hsu and Jensen, 2010). Additional expression of Lgl2 could be detected in the OPL, where it co-localized with fluorescently labelled wheat germ agglutinin (WGA), a lectin that stains the OPL (Hicks and Molday, 1985; Kivela and Tarkkanen, 1987) (Fig. 1G–G″) or with Zpr1 (Fig. 1H–H″), a marker for the cell bodies of double cones (Larison and Bremiller, 1990). Furthermore, Lgl2 localizes basolaterally in cells of the RPE, as shown by co-staining with Zpr2, which marks the RPE cell bodies (Yazulla and Studholme, 2001) (Fig. 1I–I″). These results show that Lgl2 expression is temporally and spatially controlled in the developing zebrafish retina. The expression is upregulated by 72 hpf in PRCs and RPE cells, where it is restricted to the basolateral compartment.

### Eyes of *pen/lgl2* mutant larvae are reduced in size

**Fig. 2.**
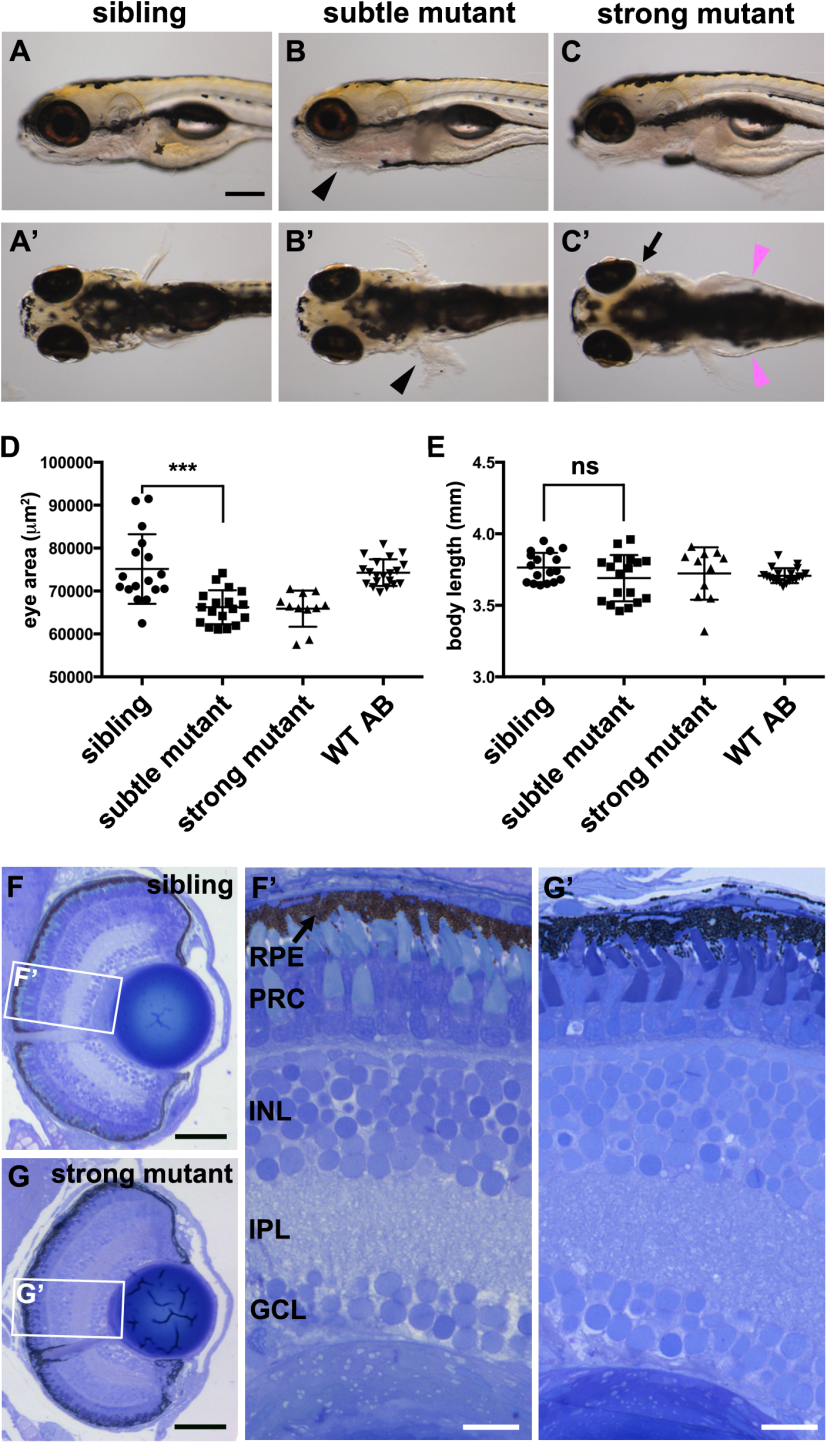
***pen/lgl2* larvae have smaller but morphologically normal eyes at 5** **dpf.** (A–C′) *pen/lgl2* eye phenotype at 5 dpf. Panels show examples of sibling (A,A′), subtle mutant (B,B′) and strong mutant (C,C′) phenotypes. Mutants were scored based on skin phenotype (B,B′, black arrowheads). *pen/lgl2* mutants with strong phenotype display severe edema (C′, magenta arrowheads) and detachment of the eyes (C′, arrow). Scale bar: 250 µm. (D,E) Measurements of lateral eye surface area (D) and body length (E) at 5 dpf in WT (*n*=20), *pen/lgl2* sibling (*n*=17), subtle (*n*=18) and strong (*n*=11) phenotype mutant larvae. *pen/lgl2* mutants display a significantly smaller eye size in comparison to sibling larvae. Graphs show mean±s.d. ***P=0.0002; ns, not significant (*P*=0.1148) by *t*-test (unpaired, with equal s.d., two-tailed). (F–G′) Toluidine Blue staining of transverse histological sections shows that, in comparison to siblings (F,F′), *pen/lgl2* strong mutant retinas laminate normally (G,G′). RPE, retinal pigment epithelium; PRC, photoreceptor cell layer; INL, inner nuclear layer; IPL, inner plexiform layer; GCL, ganglion cell layer. Scale bars: (F,G) 50 µm; (F′,G′) 10 µm.

The enhanced expression of Lgl2 in PRCs and the RPE motivated us to analyze the consequences of Lgl2 loss during retinal development. *Z*ebrafish larvae homozygous for the *lgl2* mutant allele *penner* (*pen*) exhibit a strong skin phenotype at 4.5–5 dpf (Fig. 2A–C′) (Sonawane et al., 2005). *pen/lgl2* larvae survive till 6 dpf, which allowed us to study retinal differentiation in the mutant. For the purpose of this study, the phenotypes of the mutants were divided into two classes, ‘subtle’ (Fig. 2B,B′) and ‘strong’ (Fig. 2C,C′). *pen/lgl2* larvae developing the subtle phenotype have normal overall morphology, but exhibit the known skin phenotype, characterized by hyperproliferation of the epidermis ventrally and in fin folds (Fig. 2B,B′, black arrowheads) (Sonawane et al., 2005). Larvae with a strong mutant phenotype display the same skin phenotype, but additionally show severe edema (Fig. 2C, magenta arrowheads) and ocular detachment (visible by a space around the pigmented eye, Fig. 2C, arrow). In both classes, the overall body length was not affected (Fig. 2E). Unless otherwise mentioned, larvae with subtle phenotypes were used in the following analysis.

Similar to morpholino (MO)-mediated knockdown of Lgl2 (Tay et al., 2013), loss of *pen/lgl2* results in smaller eyes. To quantify this phenotype, we measured the eye size in mutant *pen/lgl2* larvae at 5 dpf and compared it to that of siblings (wild-type and heterozygous *pen/lgl2^+/−^*). At this stage, *pen/lgl2* mutant larvae can be clearly identified based on the epidermal phenotype. In *pen/lgl2* mutant larvae, eye size, measured as pigmented area in images taken from lateral views of the larvae, is significantly decreased by 11.9% in comparison to that of siblings of the same body length (Fig. 2D). Given the reduced size of the eye, we analyzed the organization of the retina in *pen/lgl2* mutant larvae at 5 dpf. *pen/lgl2* retinas laminate normally, and all retinal layers are present in mutant eyes (compare Fig. 2F,G and F′,G′). Thus, loss of zygotic *pen/lgl2* leads to a slight but significant decrease in eye size but does not interfere with the overall stratified organization of the retina.

### Loss of zygotic *pen*/*lgl2* function does not affect photoreceptor cell polarity at 5 dpf

**Fig. 3.**
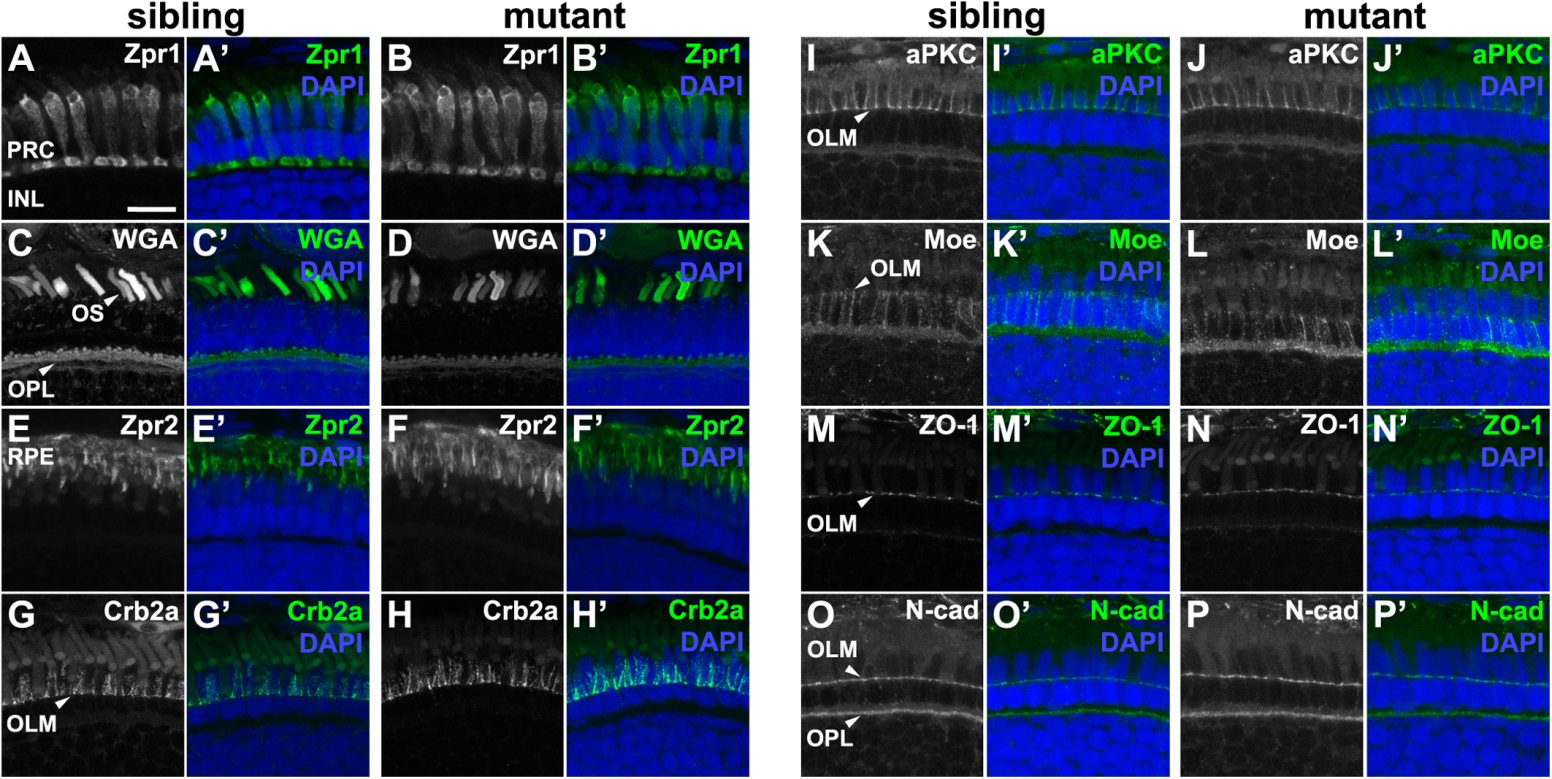
***pen/lgl2* mutant distal retina displays no significant abnormalities in polarity or cellular morphology.** Immunostaining of transverse retinal sections at 5 dpf. (A,C,E,G,I,K,M,O) Sibling and (B,D,F,H,J,L,N,P) mutant fish. (A–B′) Zpr1, (C–D′) WGA, (E–F′) Zpr2, (G–H′) Crb2a, (I–J′) aPKC, (K–L′) Moe, (M–N′) ZO-1 and (O–P′) N-cadherin immunostaining of distal retina. PRC, photoreceptor layer; INL, inner nuclear layer; OS, outer segment; OPL, outer plexiform layer; RPE, retinal pigment layer; OLM, outer limiting membrane. Scale bar: 10 µm.

Given the expression of Lgl2 in the retina and the RPE, and the observation that apico-basal polarity is affected in many epithelia lacking Lgl, we analyzed PRC morphology and polarity in eyes of *pen/lgl2* mutant larvae and their stage-matched siblings at 5 dpf by immunohistochemistry (Fig. 3). No effect on double cone morphology, or size and shape of the OS was observed in *pen/lgl2* mutant larvae as revealed by anti-Zpr1 (Fig. 3A–B′) and WGA (Fig. 3C–D′) staining, respectively. Anti-Zpr2 staining, which highlights the cell bodies of RPE cells, revealed normally shaped RPE cells in *pen/lgl2* mutant larvae. In addition, RPE cell apical protrusions seem to form and extend to the level of the OLM in *pen/lgl2* mutant larvae as in sibling controls (Fig. 3E–F′). To study PRC polarity, we used antibodies against Crb2a (Fig. 3G–H′) and aPKC (Fig. 3I–J′), which detect the subapical part of the inner segment (Horne-Badovinac et al., 2001; Hsu and Jensen, 2010; Krock and Perkins, 2014), and an antibody against Moe (Mosaic eyes, Yurt; Fig. 3K–L′), to label the basolateral membrane (Hsu et al., 2006). None of these markers were affected in *pen/lgl2* mutant larvae. Similarly, proteins at the OLM, which mark the adherens junctions, including ZO-1 (Fig. 3M–N′), N-cadherin (Fig. 3O–P′) and F-actin (data not shown) did not show any difference in localization in mutants when compared to the retina of siblings. These results show that zygotic loss of Lgl2 does not have a major effect on apico-basal polarity or junctions in PRCs at 5 dpf. This conclusion was confirmed by transmission electron microscopic analysis (data not shown).

It is unlikely that residual Lgl2 protein in *pen/lgl2* mutant retinas at 5 dpf is responsible for the lack of any mutant phenotype, since Lgl2 is significantly downregulated at this stage (Fig. S1A–G′). We also investigated the possibility that upregulation of *lgl1* compensates for the loss of *pen/lgl2*. To assess the upregulation, qRT-PCR analysis from RNA extracts of 5 dpf whole larvae was performed. On the contrary to our prediction, we found that normalized delta C_t_ values of mutant samples were higher than in siblings, indicating a downregulation of *lgl1* in *pen/lgl2* mutants (Fig. S1H). Similarly, Lgl1 protein was not significantly upregulated in retinal sections immunostained with anti-Lgl1 (data not shown). Yet, we cannot completely exclude a possible compensatory effect from Lgl1. Taken together, the lack of any obvious mutant phenotype in the retina of *pen/lgl2* mutant larvae suggests that zygotic Lgl2 is dispensable for PRC or RPE cell differentiation until 5 dpf.

**Fig. 4.**
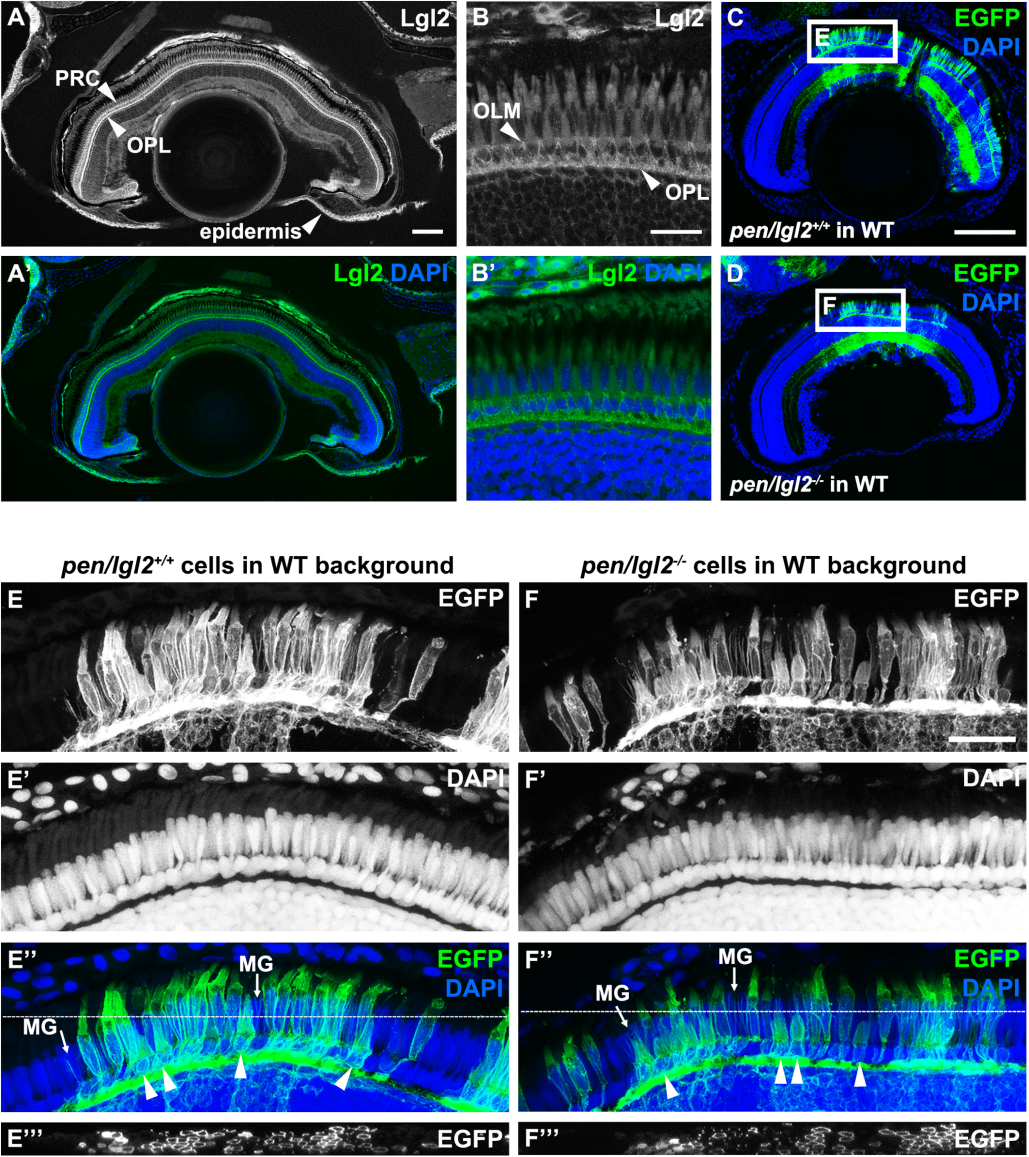
**Lgl2 expression persists in juvenile fish but endogenous zygotic Lgl2 expression is not required for PRC survival.** (A–B′) Lgl2 expression in a juvenile [10.6 mm standard length (SL)] retina. (A,A′) Lgl2 expression is detected in the epidermis and the distal retina of juvenile fish. Scale bar: 100 µm. (B,B′) Lgl2 localizes basolaterally in juvenile photoreceptors. RPE expression could not be analyzed due to pigmentation. Scale bar: 20 µm. PRC, photoreceptor cells; OLM, outer limiting membrane; OPL, outer plexiform layer. (C–F′′′) Immunostaining of transverse retinal sections of juvenile chimeric fish. EGFP-labelled sibling *pen/lgl2^+/+^* (C, 5.5 mm SL) or EGFP-labelled mutant *pen/lgl2^−/−^* (D, 5.4 mm SL) cells were transplanted into WT hosts and analyzed at 4 weeks. (C,D) Overview of the eye shows location of the transplanted EGFP+ cells in the retina. Boxed regions are shown in E–F′′′. Scale bar: 100 µm. (E–F′′′) Transplanted WT (E–E′′′) or *pen/lgl2* mutant (F–F′′′) cells (marked by EGFP) differentiate into various retinal cell types. Different types of cones can be distinguished by cell size. In E″ and F″, arrowheads denote UV cones and MG labels Müller glia processes. White lines in E″ and F″ mark the level of the orthogonal view shown in panels E′′′ and F′′′. Scale bar: 20 µm.

To find out whether Lgl2 plays a role in the retina at later developmental stages, we first analyzed the expression pattern of Lgl2 in the zebrafish retina at juvenile stages. In 4-week old fish retinas, Lgl2 expression pattern is the same as in larvae, with strong expression in the OPL and in PRCs, where it is restricted to the basolateral domain (Fig. 4A–B′). Since *pen/lgl2* mutants die by 6 dpf (Sonawane et al., 2005), we could not analyze the mutant retina at later stages. Therefore, we generated chimeric larvae by transplanting fluorescently labelled *pen/lgl2* mutant cells into WT background. The morphology of PRC clones was analyzed at 4 weeks, using transplanted fluorescently labelled *pen/lgl2^+/+^* sibling cells in WT background as controls (Fig. 4C,D). Our data show that *pen/lgl2*^−/−^ clones persist in WT retinas and develop into PRCs with normal morphology. In these clones, different types of cones can be distinguished based on their cell size (Fig. 4E″,F″, arrowheads denote UV cones). Processes formed by MG cells are evident as well (Fig. 4E″,F″, arrows). Analysis of labelled clusters in Z-axis suggests that mutant PRCs surrounded by other mutant cells (no WT contact) have normal morphology (Fig. 4E′′′,F′′′). To conclude, our data show that zygotic Lgl2 is not necessary for PRC maturation and survival during larval and juvenile stages.

### Knockdown of maternal and zygotic Lgl2 leads to disorganization of PRC layering

*pen/lgl2* has been reported to be maternally provided (Sonawane et al., 2005; Tay et al., 2013). It is possible that Lgl2 derived from maternal transcripts is still present during the period of photoreceptor differentiation in the retina. Therefore, we asked whether the maternal component of Lgl2 compensates for the lack of zygotic Lgl2 in the early stages of retinal development.

**Fig. 5.**
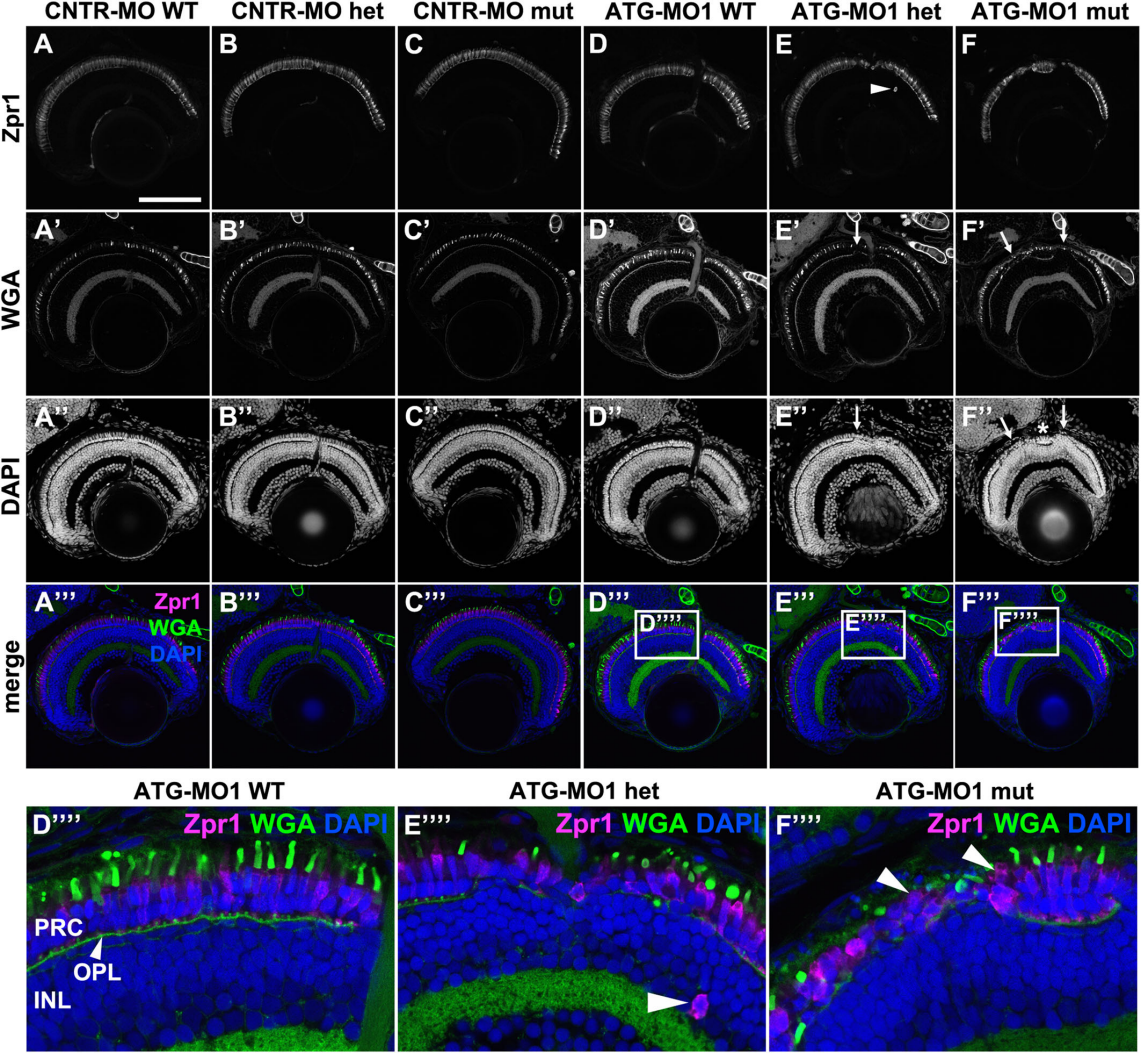
**Knockdown of Lgl2 in *pen/lgl2* clutches leads to disorganization of retinal lamination.** Immunostaining of transverse retinal sections at 5 dpf of MO-injected and genotyped *pen/lgl2* clutches. (A–C″′) CNTR-MO-injected fish and (D-F′′′′) ATG-MO1-injected fish. Zpr1 (A–F) stains double cone cell bodies and WGA (A′–F′) the OPL and OSs. In ATG-MO1-injected hetero- (E–E′′′′) or homozygous (F–F′′′′) fish, layering in the distal retina is disturbed. Arrows in E′, E″, F′ and F″ mark disorganized clusters separated by normally aligned cells (asterisk). Boxed areas in D′′′–F′′′ show enlarged views of PRCs in MO-injected *pen/lgl2* WT (D′′′′), heterozygote (E′′′′) and mutant (F′′′′) fish. Occasionally Zpr1 positive cells were seen outside of the PRC layer (E,E′′′′, arrowheads). Arrowheads in F′′′′ mark apically displaced PRCs that are localized on top of another PRC. PRC, photoreceptor layer; OPL, outer plexiform layer; INL, inner nuclear layer. Scale bar: 100 µm.

To address this question, we knocked down early Lgl2 protein expression by injecting MOs against *pen/lgl2* into one-cell stage embryos. We used two ATG-blocking MOs which have been previously documented to cause specific phenotypes in other zebrafish tissues [ATG-MO1 (Dodd et al., 2009; Hava et al., 2009; Sonawane et al., 2005, 2009; Tay et al., 2013; Westcot et al., 2015) and ATG-MO2 (Hava et al., 2009; Tay et al., 2013)] (Fig. S2). These MOs work effectively in the retina, since a clear decrease in Lgl2 staining was observed upon their injections into wild-type embryos (Fig. S2F–K′). Knockdown of Lgl2 using a lower ATG-MO1 concentration (2.5 ng per embryo) also leads to decreased Lgl2 staining (Fig. S2F–G′) but does not yield any abnormal overall embryonic (Fig. S2A–E) or retinal phenotypes (Fig. S2F–G′). In contrast, the same concentration of ATG-MO2-injected into wild-type induced defects in overall morphology (Fig. S2E). However, when either ATG-MO1 or ATG-MO2 was injected into the *pen/lgl2* background at this low concentration, there was an obvious effect on retinal lamination in both cases (Fig. 5 and Fig. S3). Therefore, we used the ATG-MO1 for further experiments.

Closer inspection of affected retinas of genotyped fish revealed PRC layering defects in *pen/lgl2* homo- and heterozygous background at 5 dpf upon *pen/lgl2* knockdown (Fig. 5E-F′′′). This phenotype was very rarely observed in wild-type siblings. The defects were seen in five out of six homozygotes, eight of eleven heterozygotes and one of six wild-type fish injected with ATG-MO1 and genotyped at 5 dpf. The phenotype was mostly found in the central retina and was manifested by the formation of disorganized cell clusters (Fig. 5E″,F″, arrows), which were often separated by properly organized tissue (Fig. 5F″, asterisk). In these abnormal cell clusters, the alignment of PRCs becomes irregular (Fig. 5F′′′, arrowheads). In addition, the OPL is interrupted (Fig. 5E′,F′, arrows). Occasionally, PRCs come to lie in the other nuclear layers of the retina (Fig. 5E′′′). This phenotype was never observed in *pen/lgl2* mutants without knockdown of maternal Lgl2. From this, we conclude that a threshold level of Lgl2 is required for normal retinal development.

**Fig. 6.**
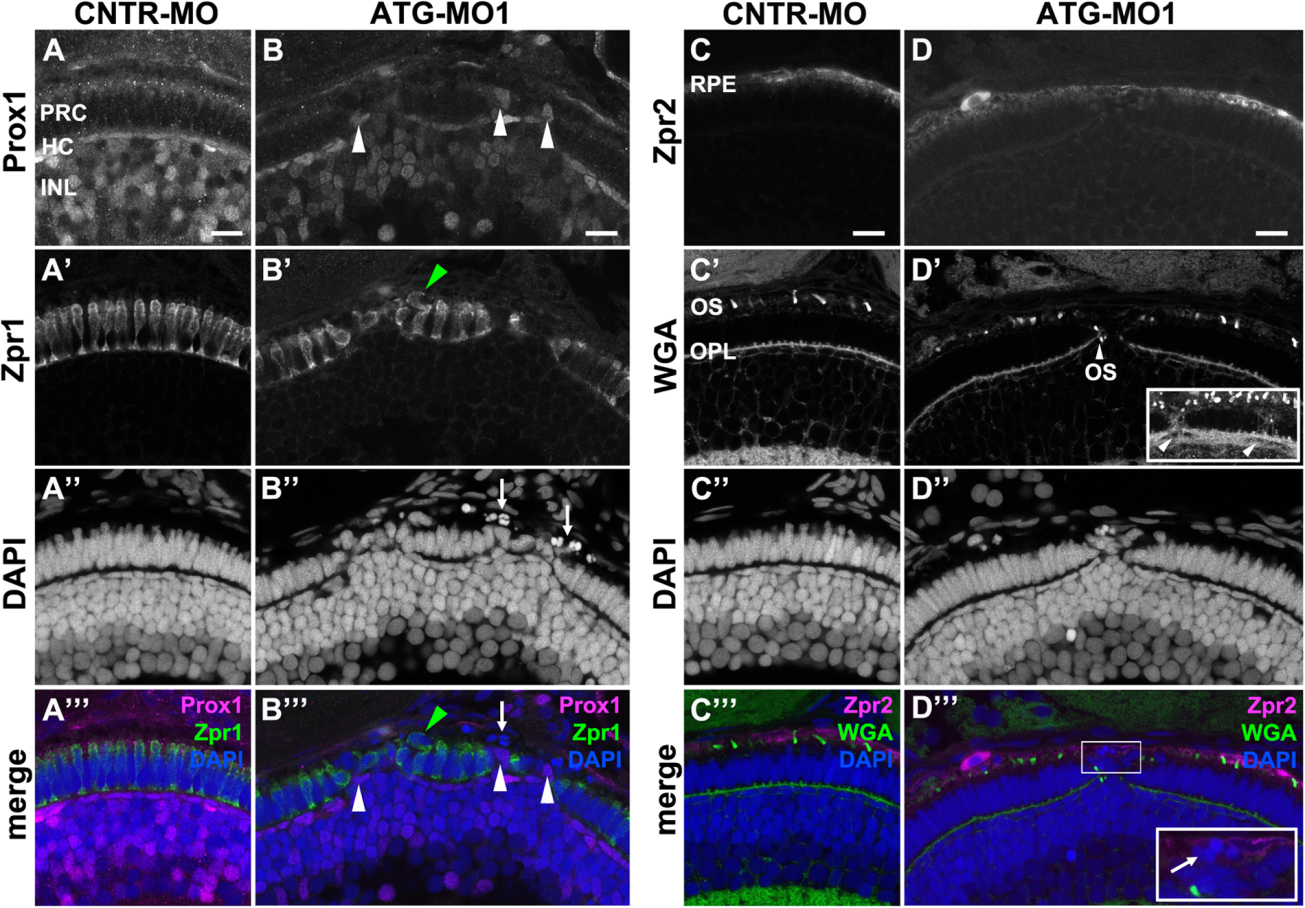
**Knockdown of Lgl2 in *pen/lgl2* clutches affects the organization of the outer and inner nuclear layers.** Immunostaining of transverse retinal sections at 3 dpf of MO-injected *pen/lgl2* clutches. (A–A′′′,C–C′′′) *pen/lgl2* clutch injected with CNTR-MO and (B–B′′′,D–D′′′) *pen/lgl2* clutch injected with ATG-MO1. (A–B′′′) Prox1- (A,B) and Zpr1-(A′,B′) positive cells are well separated from each other in control retinas (A–A′′′) but mix in Lgl2 morphants (B–B′′′). White arrowheads in B and B′′′ denote Prox1-positive cells that have been apically displaced. Green arrowheads in B′ and B′′′ mark an apically displaced PRC. Arrows in B″ and B′′′ mark pyknotic nuclei apical to the photoreceptor layer. (C–D′′′) Zpr2-positive RPE cells (C,D) in MO-injected *pen/lgl2* clutches (D–D′′′) surround nuclei apical to the PRC layer. WGA staining (C′,D′) shows that in Lgl2 morphants, disorganized PRCs have outer segments (OS), but these can be found next to the OPL (arrowhead in D′). Projection of multiple optical planes shows breakages in the plane of OPL (inset in D′, arrowheads). Inset in D′′′ shows pyknotic nuclei (arrow) that appear to be surrounded by the Zpr2 signal. PRC, photoreceptor cells; HC, horizontal cells; INL, inner nuclear layer; OPL, outer plexiform layer. Scale bars: 10 µm.

**Fig. 7.**
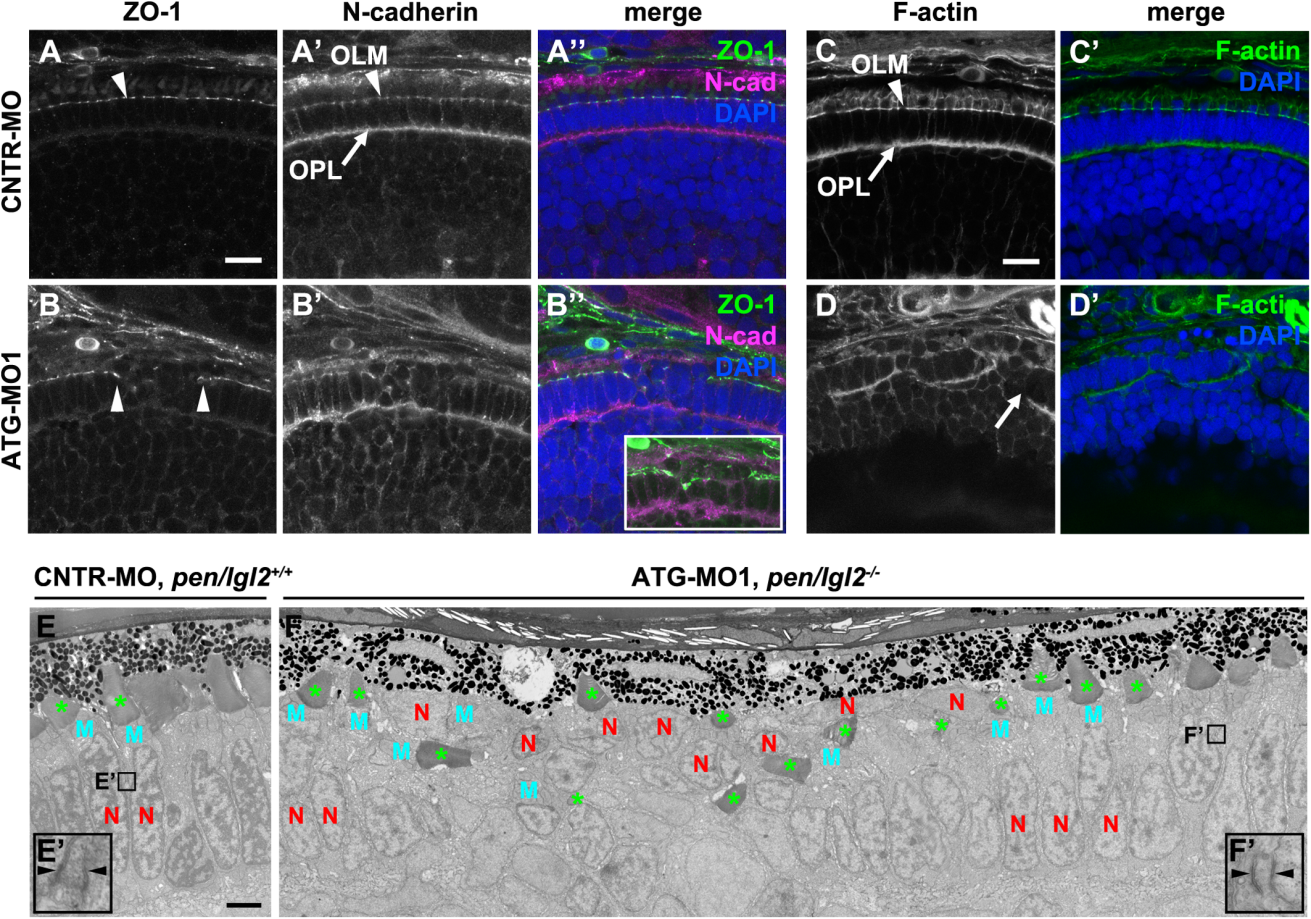
**Knockdown of Lgl2 in *pen/lgl2* clutches leads to OLM and OPL abnormalities.** Immunostaining (A–D′) and TEM (E–F′) of transverse retinal sections at 3 dpf of MO-injected *pen/lgl2* clutches. (A–A″, C,C′) *pen/lgl2* clutch injected with CNTR-MO and (B–B″,D,D′) *pen/lgl2* clutch injected with ATG-MO1. ZO-1 (A,B), N-cadherin (A′,B′) and F-actin (C,D) localization to OLM (arrowheads) and OPL (arrows) is abnormal in morphants. Inset in B″ shows a projection of 15 optical planes in which discontinuity of the OLM is evident. Scale bars: 10 µm. (E,E′) *pen/lgl2^+/+^* sibling injected with CNTR-MO shows a single, aligned layer of PRCs. E′ shows a higher magnification of a PRC adherens junction (arrowheads). (F,F′) *pen/lgl2* mutant injected with ATG-MO1. PRCs have differentiated outer segments (green asterisks) but are disorganized. In the cells next to the disorganized area, normal adherens junctions are detected (F′, arrowheads). N, nucleus; M, mitochondria. Scale bar: 2 µm.

To corroborate these findings, we injected ATG-MO1 into the progeny of a *pen/lgl2^+/−^* incross and analyzed the retina of these clutches (from here on referred to as ‘*pen/lgl2* clutches’). Staining with Zpr1 and Prox1 antibodies to identify double cones and INL cells, respectively, revealed defects in retinal layering already at 3 dpf (Fig. 6A–B′′′). In control MO-injected *pen/lgl2* clutches at 3 dpf, Prox1 labelled a subset of nuclei in the INL (Vihtelic et al., 2001). Prox1-positive nuclei were well separated from the PRC layer, marked by Zpr1 (Fig. 6A–A′′′). In contrast, the separation of the INL and the PRC layer is incomplete in the retina of ATG-MO1-injected embryos, so that PRCs and cells from the INL are found next to each other, with INL cells displaced apically towards the PRC layer (Fig. 6B,B′′′, white arrowheads). Double cones are no longer restricted to a single layer, which becomes obvious by the fact that some PRCs are displaced apically and can be found in the space between the PRC layer and the RPE cells (Fig. 6B′,B′′′, green arrowhead). Pyknotic nuclei were identified within the RPE by very bright DAPI signals within Zpr2-positive RPE cells, suggesting dying cells (Fig. 6D′′′, inset, arrow). Occasionally, Zpr1-positive cells were also seen displaced basally and appeared within the other nuclear layers of the retina (Fig. 5E,E′′′, white arrowhead). Strikingly, apically or basally displaced PRCs differentiate and form OSs, detected by WGA staining (Fig. 6D′, arrowhead) and by transmission electron microscopy (TEM) (Fig. 7F). Taken together, the retinal phenotype observed upon knockdown of maternal and zygotic Lgl2 allows the conclusion that loss of Lgl2 impairs the integrity of the PRC layer, resulting in apical and basal displacement of PRCs.

### Lgl2 downregulation causes discontinuities in the OLM and the OPL

To further understand how loss of Lgl2 impairs the organization of the PRC layer, we analyzed the integrity of the OLM as well as the OPL. At 3 dpf, ZO-1 marks the OLM, a junction that facilitates the adhesion between PRCs and between PRCs and MG cells. In *pen/lgl2* clutches injected with control MO, a continuous ZO-1 staining can be seen throughout the PRC layer (Fig. 7A). In contrast, ATG-MO1 injection induced clusters of disorganized cells, in which the continuous ZO-1 staining is interrupted (Fig. 7B, arrowheads). Similar defects are observed in N-cadherin (Fig. 7A′,B′) and F-actin (Fig. 7C,D) distribution, both of which are interrupted in ATG-MO1 induced cell clusters, while they are continuous in the OLM in areas with wild-type appearance.

N-cadherin forms a continuous layer also in the OPL, basal to the PRCs (Fig. 7A′, arrow). This layer is interrupted in disorganized retinal regions of embryos injected with ATG-MO1 (Fig. 7B′). WGA staining clearly illustrates the breakdown of the OPL, as holes are visible within the plane of the OPL in a projection of confocal sections (Fig. 6D′, inset). However, it should be noted that in areas outside of the clusters the localization of ZO-1, actin and N-cadherin as well as the basal WGA-staining of the OPL is normal. TEM analyses confirm that junctions appear normal in cells next to disorganized clusters (Fig. 7F′), but the presence or absence of intact junctions within clusters cannot be confirmed due to the disorganization of the tissue. These results show that a threshold level of Lgl2 is required for the integrity of the OLM and the OPL in order to maintain the stratified organization of the distal retina.

## DISCUSSION

In vertebrates, proper vision depends on the correct specification and differentiation of several neuronal cell types, the formation of synaptic contacts between them and their organization into a highly stratified retina (Amini et al., 2017; Baier, 2013; Morgan and Wong, 1995). The visual process is initiated by PRCs, rods and cones, which capture photons of light by photosensitive pigments. The activation of cone and rod visual pigments triggers the phototransduction cascade, which ultimately transmits the signal into the brain. PRCs are highly polarized cells, forming an apical, light-sensitive organelle, called the OS, and the basal ribbon synapse, which connects the PRCs to second order neurons (horizontal and bipolar cells). To develop these features, PRCs have to establish and maintain apico-basal polarity and to form adhesive contacts, a prerequisite for layer formation. Both processes are tightly coupled and loss of either is linked to the breakdown of the layered structure, retinal degeneration and ultimately blindness. In human, several retinopathies such as retinitis pigmentosa (RP), Leber's congenital amaurosis (LCA) or Usher syndrome are associated with loss of function of genes regulating polarity or adhesion [reviewed in (Chacon-Camacho and Zenteno, 2015; El-Amraoui and Petit, 2010; Verbakel et al., 2018; Yan and Liu, 2010)]. Therefore, the establishment and maintenance of apico-basal polarity and formation of cellular junctions is crucial for a functional retina. Here we show that knockdown of both maternal and zygotic Lgl2, a protein known to regulate apico-basal polarity in various epithelia, results in the disorganization of the PRC layer. These findings add to our knowledge of the molecular mechanisms regulating morphogenesis of PRCs and may help in understanding their dysregulation in disease.

The retina of *pen/lgl2* homozygous mutant zebrafish larvae derived from heterozygous animals laminates and differentiates normally, demonstrating that maternal Lgl2 is sufficient for the development of the embryonic and larval retina. This is different from its requirement in the epidermis, where already the loss of zygotic gene function results in severe defects due to impaired hemidesmosome formation, followed by loss of cell-matrix contacts in the basal layer of the epidermis, blistering and hyperproliferation (Sonawane et al., 2005, 2009). Since the complete MO-induced knockdown (KD) of *pen/lgl2* causes severe overall phenotypes (Tay et al., 2013), we injected lower MO concentrations into the *pen/lgl2* background. This leads to abnormal organization of the PRC layer in the retina of hetero- and homozygous mutant larvae. From these data we concluded that the maternal contribution of gene expression is sufficient to allow normal retinogenesis, and that the loss of one or two functional copies of *pen/lgl2* provides a sensitized genetic background, which allows study into the role of *pen/lgl2* in retinal development.

Lgl proteins are well known for their function in establishment and maintenance of apico-basal polarity and junctional complexes in epithelial cells (Cao et al., 2015; Grifoni et al., 2013). Loss of Lgl function has tissue-specific consequences: in some cell types polarity is strongly affected (Bilder et al., 2000; Klezovitch et al., 2004), while in others Lgl is required for junctional integrity and adhesion (Jossin et al., 2017; Sonawane et al., 2005, 2009; Tay et al., 2013). In vertebrates, the situation is complicated by the fact that the genomes encode two orthologues, *lgl1* and *lgl2*. Zebrafish *lgl1*, for example, plays a role in apical differentiation in the pseudostratified retinal neuroepithelium. Cells with reduced Lgl1 levels retain junctions and overall epithelial integrity, but develop an enlarged apical membrane. This results in increased Notch signaling and, as a consequence, impaired neurogenesis (Clark et al., 2012). Deletion of mouse *Llgl1* specifically in embryonic cortical neural stem cells affects the integrity of the apical junctional complex (AJC). Loss of AJC integrity is likely due to impaired interactions between LLGL1 and N-cadherin, which is followed by mislocalization of N-cadherin. Affected neural stem cells are internalized and form rosette-like structures, while their overall apico-basal polarity is retained. Ultimately, this leads to ectopic formation of neurons at the ventricular surface (Jossin et al., 2017). Overall apico-basal polarity is also not affected in the cells of the zebrafish Kuppfer's vesicle in Lgl2 morphant embryos (Tay et al., 2013), or in basal epidermal cells in *pen/lgl2* mutant fish (Sonawane et al., 2005), but both cell types display defects in cellular adhesion upon the loss of Lgl2.

As shown here, loss of zygotic *pen/lgl2* activity does not induce any gross modifications of apico-basal polarity and compartment size in PRCs of the larval retina. The inner and OSs develop normally and are similar in size to those of controls. Similarly, no major defects in PRC morphology could be detected upon additional knockdown of the maternal component of *pen/lgl2* gene expression, indicating that overall apico-basal polarity is not affected. However, KD of both maternal and zygotic *pen/lgl2* function results in defects in the organization of the PRC layer, most likely due to impaired adhesion. This assumption is corroborated by impaired N-cadherin localization at the OLM and the concomitant loss of actin and ZO-1 upon combined deficiency in maternal and zygotic *pen/lgl2* function. This phenotype is strikingly similar to that observed upon *Llgl1* knockdown in mouse embryonic cortical neural stem cells (Jossin et al., 2017). Furthermore, the retinal disorganization caused by the lack of Lgl2 resembles the loss of N-cadherin (*pac^rw95^* mutants) in the zebrafish retina, which results in lamination defects without affecting neuronal differentiation (Masai et al., 2003). In Lgl2 morphant Kuppfer's vesicles, basolateral transport of E-cadherin is abnormal (Tay et al., 2013), and similarly, the loss of *pen/lgl2* in the basal epidermis causes defects in hemidesmosome formation due to impaired delivery of integrin alpha 6 (Itga6) (Sonawane et al., 2009). These results demonstrate a role for Lgls in regulating cell adhesion via polarized trafficking, and future experiments should address the role of Lgl2 in regulating the dynamics of adherens junction components in the distal retina.

*lgl* was originally discovered as tumor suppressor gene (Bilder, 2004; Gateff, 1978) and can regulate spindle orientation and asymmetric cell division (Bell et al., 2015; Betschinger et al., 2003; Carvalho et al., 2015; Wirtz-Peitz et al., 2008; Yasumi et al., 2005). In the wild-type zebrafish retina committed photoreceptor precursor cells are dividing parallel to the tissue layer between 60–72 hpf (Suzuki et al., 2013; Weber et al., 2014). Therefore, loss of PRC layering observed in *pen/lgl2* MO-injected fish could be the result of hyperproliferation and/or misoriented mitotic spindles, which would place cells outside the plane of the PRC layer, between the PRC layer and the pigment epithelium. Preliminary data on proliferation in the retina revealed no significant difference in the number of phospho-histone H3 positive mitotic cells at 48–72 hpf in morphant versus control retinas in *pen/lgl2* clutches (data not shown). Only occasionally dividing cells were found within disorganized cell clusters, indicating that aberrant cell divisions are unlikely to be the cause for the formation of cell clusters. Finally, no cell divisions were detected in the retina of Lgl2 morphants at stages at which proliferation has ceased in the central retina (5 dpf). This is consistent with observations made in Kuppfer's vesicle upon KD of *pen/lgl2*, in which no change in proliferation was detected (Tay et al., 2013), but is in marked contrast to those made in the mouse brain neuroepithelium or in the zebrafish epidermis, where loss of Lgl2 leads to hyperproliferation (Klezovitch et al., 2004; Reischauer et al., 2009). In addition, hyperproliferating *pen/lgl2* mutant epidermal cells undergo EMT and acquire migratory potential (Reischauer et al., 2009). In *pen/lgl2* morphant retinas, PRCs were occasionally found in more basal layers, e.g. in the INL, but their number was very low. Therefore, we favor the conclusion that the major cause for the retinal phenotype upon loss of Lgl2 is the lack of adhesion in the PRC layer.

The detection of disorganized PRC clusters at 3 dpf upon KD of Lgl2 in *pen/lgl2* clutches suggests a function for Lgl2 either in organizing the PRC layer during its lamination or at later stages during the maturation of the photoreceptor cell layer. Preliminary data show that retinas of Lgl2 MO-injected *pen/lgl2* clutches appear normal at 48 and 54 hpf, and clear signs of disorganization and cell cluster formation appeared in embryos older than 60 hpf. It has to be pointed out, however, that identifying a disorganized PRC layer prior to 60 hpf is challenging, as it is only at this time that the cell layer appears straight and clearly separated from the INL. Therefore, we cannot exclude that the phenotype emerges already during lamination of the PRC layer. However, nuclei/cells mis-localized above the PRC layer were never observed during early stages of development (48–54 hpf), but only accompanied disorganized PRC clusters starting at 60 hpf. Future analyses, including live imaging of fluorescently labelled PRCs to visualize cell morphological and junctional aberrations, are needed to track the onset of cluster formation.

Why are only groups of cells affected by the KD of *pen/lgl2*, rather than the entire retina? Two scenarios can explain this conundrum. First, variation in MO concentration within retinal cells could induce variable degrees of *pen/lgl2* knockdown. Maternal-zygotic *pen/lgl2* mutants should be generated to determine the effects of complete Lgl2 loss on the retina, but this was out of the scope of our study. Alternatively, mutant cell clusters could be caused by a defect in one of the cell types that interact with PRCs. For example, the apical processes of MG cells interact with PRCs and form an integral part of the OLM (Gosens et al., 2008; Wei et al., 2006; Zou et al., 2012). Preliminary data from retinas of embryos expressing the MG-specific reporter GFAP:EGFP (Bernardos and Raymond, 2006), injected with Lgl2 MO show loss of apical MG processes within a phenotypical cluster. In addition, cell bodies of some MG are mis-localized basally into the GC layer (data not shown). Whether these defects in MG cells are the cause or the consequence of PRC cluster formation has to be elucidated in the future. Furthermore, our data show strong expression of Lgl2 in cells of the RPE. Defects in the RPE are frequently associated with PRC abnormalities, including degeneration, since OS maintenance depends on a functional RPE (Kevany and Palczewski, 2010; Sparrow et al., 2010; Strauss, 2005). However, we did not detect any gross changes in RPE cell morphology or ultrastructure. Yet, we cannot exclude a role for *pen/lgl2* in the RPE, which would impact on the organization of the PRC layer. In the future, cell-type specific inactivation of *pen/lgl2* would help to identify in which cell type this gene is required.

## MATERIALS AND METHODS

### Zebrafish strains, transgenic lines and husbandry

Adult zebrafish (*Danio rerio*) were maintained at 26.5°C with a 10/14 h dark/light cycle. Embryos and larvae were raised at 28.5°C and staged according to Kimmel et al. (1995) and Parichy et al. (2009). For the experiments in wild-type fish, the AB strain was used. The *pen/lgl2* mutant line has been described before (Sonawane et al., 2005). For transplantation experiments, *pen/lgl2* was crossed into the Tg(*bactin*:mRas-EGFP) line (Cooper et al., 2005). All animal experiments were performed in accordance with the German Animal Welfare Act and the EU directive 2011/63/EU.

### *pen/lgl2* genotyping

For genotyping the *pen/lgl2* fish, part of the *lgl2* locus containing the previously characterized point mutation (Sonawane et al., 2005) was amplified (forward primer: 5-AGCCATCACTTGCTCACACCAC-3; reverse primer: 5-TGCTGAAGGGAAAAAATACACATTC-3) using the EmeraldAmp MAX HS PCR master mix (Clontech). Amplification products were sequenced using the primer 5-GAAAATGCTTGTAATGTACCTGC-3.

### Imaging of *pen/lgl2* phenotypes

Larvae were anesthetized in tricaine, transferred to 2% methylcellulose and imaged with Olympus SZX16 stereoscope using QImaging MicroPublisher 5.0RTV camera with QCapture software. Body length and eye area were quantitated using the straight line and polygon tools, respectively, in Fiji (Schindelin et al., 2012). Graphs depicting the data were drawn and statistical analysis performed with GraphPad Prism software.

### Histology and TEM

For histology and TEM, heads of 5 dpf larvae were fixed in 2% glutaraldehyde, 2% paraformaldehyde in 50 mM Hepes (pH 7.25) for 1.5 h at room temperature (RT), then at 4°C overnight. Heads were washed 5×3 min in Hepes buffer, 2×5 min in PBS, incubated for 60 min in 1% OsO_4_/1.5% potassium ferrocyanide in PBS, washed 2×5 min in distilled water and dehydrated in an ethanol series. Samples were incubated 2×10 min in propidium oxide and embedded in Durcupan. For histology, semithin sections of 500 nm were stained with 1% Toluidine Blue, 0.5% sodium borate solution and mounted in Entellan (Merck). Samples were imaged with Zeiss Imager.Z1 microscope using AxioCam HRc and AxioVision software (Zeiss). For TEM, ultrathin sections of 70 nm were stained with uranyl acetate and lead citrate, and imaged with a Morgagni TEM (80 kV) using a Morada CCD camera (EMSIS GmbH) and ITEM software (EMSIS GmbH). Images were processed using TrakEM2 in Fiji (Cardona et al., 2012; Schindelin et al., 2012) and Adobe Photoshop.

### RNA isolation and qPCR

For isolation of total RNA from 5 dpf larvae, 30 larvae were pooled and homogenized in Trizol (Ambion) using a syringe (1 ml) and a needle (22 g). The following steps were done according to the manufacturer's protocol. 1 µg of total RNA was treated with DNAse I [New England Biolabs (NEB)] and transcribed to cDNA using oligo(dT)_12-18_ (Invitrogen) and random hexamer (Invitrogen) primers with the SuperScript III reverse transcriptase (Invitrogen) according to the manufacturer's protocol. To assess the expression levels of *lgl1* the following primers were used: forward 5-CGCTGTGTGGAGTGGATATAGA-3, reverse 5-CTGCTTGTGACTTGTGTGTTCC-3. *lgl1* C_t_ values were normalized to *rpl13a*, whose expression was assessed using the following primers: forward 5-TCTGGAGGACTGTAAGAGGTATGC-3, reverse 5-AGACGCACAATCTTGAGAGCAG-3 (Tang et al., 2007). qPCR reactions were set up using the FastStart Essential DNA Green master mix (Roche) and run with the LightCycler96 (Roche). Delta_Ct_ values were calculated by C_t(*lgl1)*_–C_t(*rpl13a)*_. A graph depicting the data was drawn and statistical analysis performed with GraphPad Prism software.

### Immunostaining on cryosections

For immunostaining, embryos were treated with phenylthiourea (PTU, 0.2 mM) to prevent pigmentation from 22 hpf on. Embryos or larvae were fixed with 4% PFA in PBS overnight at 4°C, washed with PBS with 0.1% Tween-20 (PBST) and processed through 10%, 20%, 30% sucrose solutions. Samples were incubated in 1:1 30% sucrose, 50% NEG-50 (Thermo Fisher Scientific) and then embedded in NEG-50 and stored at −80°C. Cryosections were generated using a Microm HM 560 (Thermo Fisher Scientific) at 16–20 µm for normal immunostaining and at 25 µm for imaging of transplanted chimeras. Sections were allowed to dry for 2 h and then stored at −20°C. Upon staining, slides were thawed for 2 h at RT and washed in PBS 2×10 min. For Lgl2, aPKC, and ZO-1 stainings antigen retrieval was done by incubating slides in 10 mM sodium citrate, 0.05% Tween-20 (pH 6) for 20 min at 70°C. Slides were allowed to cool to RT in antigen retrieval buffer for a further 30 min and were then washed 3×5 min with PBST. Samples were permeabilized with 0.1% SDS in PBS for 15 min, washed 3×5 min with PBST, blocked in 10% normal horse serum (NHS) in 0.3% Triton X-100 in PBS for a minimum of 1 h, and then stained overnight with the primary antibody in 1% BSA, 1% NHS, 0.3% Triton X-100 in PBS at 4°C. On the second day, slides were washed 6×30 min with PBST, and incubated overnight with the secondary antibody in 1% bovine serum albumin (BSA), 1% NHS, 0.3% Triton X-100 in PBS at 4°C. Primary antibodies were used at the following concentrations: Lgl2, 1:400 (Sonawane et al., 2009); ZO-1, 1:200 (Molecular Probes, #339100); Zpr1, 1:200 (ZIRC); Zpr2, 1:100 (ZIRC); Zs4/Crb2a, 1:20 (ZIRC); aPKC, 1:50 (C-20, sc-216-G, Santa Cruz Biotechnology), Moe, 1:200 [gift from Abbie Jensen (University of Massachusetts Amhurst)], Prox1, 1:100 (Millipore, #AB5475); anti-EGFP, 1:250 (Roche, #11814460001). Secondary antibodies (Alexa Fluor 488 or 568 conjugates) were used at 1:500 (Invitrogen). Alexa Fluor 488 phalloidin (1:100, Invitrogen), Alexa Fluor 488 conjugate of WGA (1:200, Molecular Probes) and DAPI (Roche) were added to the secondary antibody incubation. Slides were mounted in Vectashield (Vector Laboratories) and imaged with a Zeiss LSM 880 confocal microscope with 63×Zeiss LCI Plan-Neofluar 1.3 objective using ZEN software. Optical sections acquired were 0.35 µm. Images were processed using Fiji (Schindelin et al., 2012) and Adobe Photoshop.

### Blastula cell transplantation

For transplantations, the progeny of an incross of Tg(*bactin*:mRas-EGFP);*pen/lgl2*^+/−^ carriers was used as donors and WT AB as recipients. Early blastula stage embryos were dechorionated with pronase (0.3 mg/ml). Transplantation setup consisted of a 1 ml syringe connected to a sideport of a micromanipulator needle holder with a glass capillary needle. 20–40 blastomeres were transplanted at the sphere stage to the animal pole of the recipient embryo, which targets the transplanted cells to the future eye (Kimmel et al., 1990). The donors were raised to 2 dpf and genotyped for *pen/lgl2*, and recipients were raised till 4 weeks of age. Juvenile fish were staged according to Parichy et al. (2009), and their heads fixed and processed as described for 5 dpf larvae for immunohistochemistry.

### Morpholino knockdown

Morpholinos were injected into either to the progeny of an incross of *pen^+/−^* fish or into WT AB embryos at the one-cell stage. The following MOs were used: Lgl2 ATG-MO1 5-GCCCATGACGCCTGAACCTCTTCAT-3 (Dodd et al., 2009; Hava et al., 2009; Sonawane et al., 2009; Tay et al., 2013; Westcot et al., 2015), Lgl2 ATG-MO2 5-AGCCGGGACTCAAACTGCCCTCTCT-3 (Hava et al., 2009; Tay et al., 2013) and Lgl2 5-base mismatch CNTR-MO 5-GCACATAACGCCTCAACCTGTTAAT-3 (Sonawane et al., 2009) (Gene Tools, LLC). Morpholino concentrations were selected based on testing a series of concentrations and scoring the phenotype of the embryos (Fig. S2). Unless otherwise indicated, a bolus of 1 nl, delivering 2.5 ng of MO was injected per embryo. Bolus volume was also kept constant when the total amount of MO was changed.

## Supplementary Material

Supplementary information
