# Supplementary material for: penner/lgl2 is required for the integrity of the photoreceptor layer in the zebrafish retina
Source: Biol Open. 2019 Apr 15;8(4):bio041830. doi: 10.1242/bio.041830 (PMC6503998; doi:10.1242/bio.041830)
Supplement: Supplementary information [file biolopen-8-041830-s1.pdf]

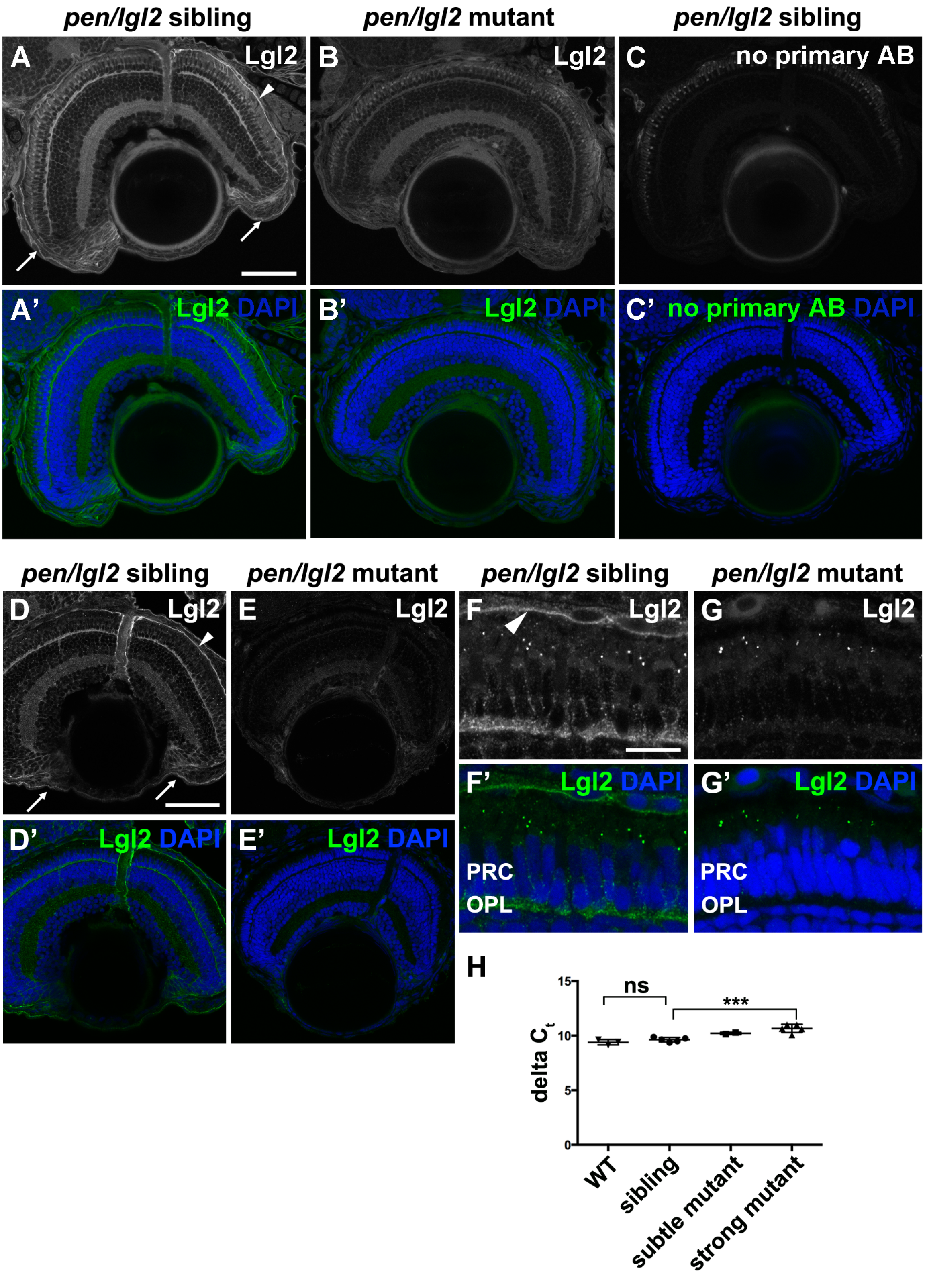

Figure S1. Lgl2 expression in *pen/lgl2* larvae. A-G'. Anti-Lgl2 immunostaining of transverse retinal sections of larvae at 5 dpf. Lgl2 protein level is strongly downregulated in *pen/lgl2* mutant eyes. **A-C'.** Anti-Lgl2 staining with antigen retrieval in *pen/lgl2* sibling (A, A') and mutant (B, B') eyes. Control (C, C') is without primary antibody to show unspecific background staining. In sibling retinas (A) Lgl2 is detected in the RPE (arrowhead), the OPL and the epidermis (arrows). In the mutant retina (B) no localized signal can be distinguished in the epidermis or the RPE. Scale bar, 50 µm. **D-G'.** Anti-Lgl2 staining without antigen retrieval in *pen/lgl2* sibling (D, F) and mutant (E, G) eyes. In retinas of siblings (D, D'), Lgl2 expression is visible in the epidermis (arrows) as well as in the RPE

Biology Open • Supplementary information

(arrowhead) and the OPL. This expression pattern is not detected in mutant retinas (E, E'). Higher magnification of the sibling distal retina (F, F') shows a clear Lgl2 signal in the RPE (arrowhead) and the basal side of PRCs, which is not present in the mutant (G, G'). A dotty, unspecific background staining is seen in the outer segment area without antigen retrieval (F-G’). Antigen retrieval gives a clear Lgl2 signal, but an overall higher background staining is observed (compare A vs. B to D vs. E). PRC, photoreceptor cells; OPL, outer plexiform layer. Scale bar D, 50 µm; F, 10 µm. **H.** *lgl1* mRNA levels are not upregulated in *pen/lgl2* larvae at 5 dpf. Graph shows mean delta Ct values of *lgl1* normalized to housekeeping gene with standard deviation in whole larval extracts from WT, *pen/lgl2* sibling and mutants. Numbers of biological replicates were as follows: WT, n=3; sibling, n=5; subtle mutant, n=2; strong mutant, n=5. All samples were run in one qPCR experiment. *** in H denotes a p-value of 0.006 and ns of 0.1987 by t-test (unpaired, with equal s.d., two-tailed).

Biology Open • Supplementary information

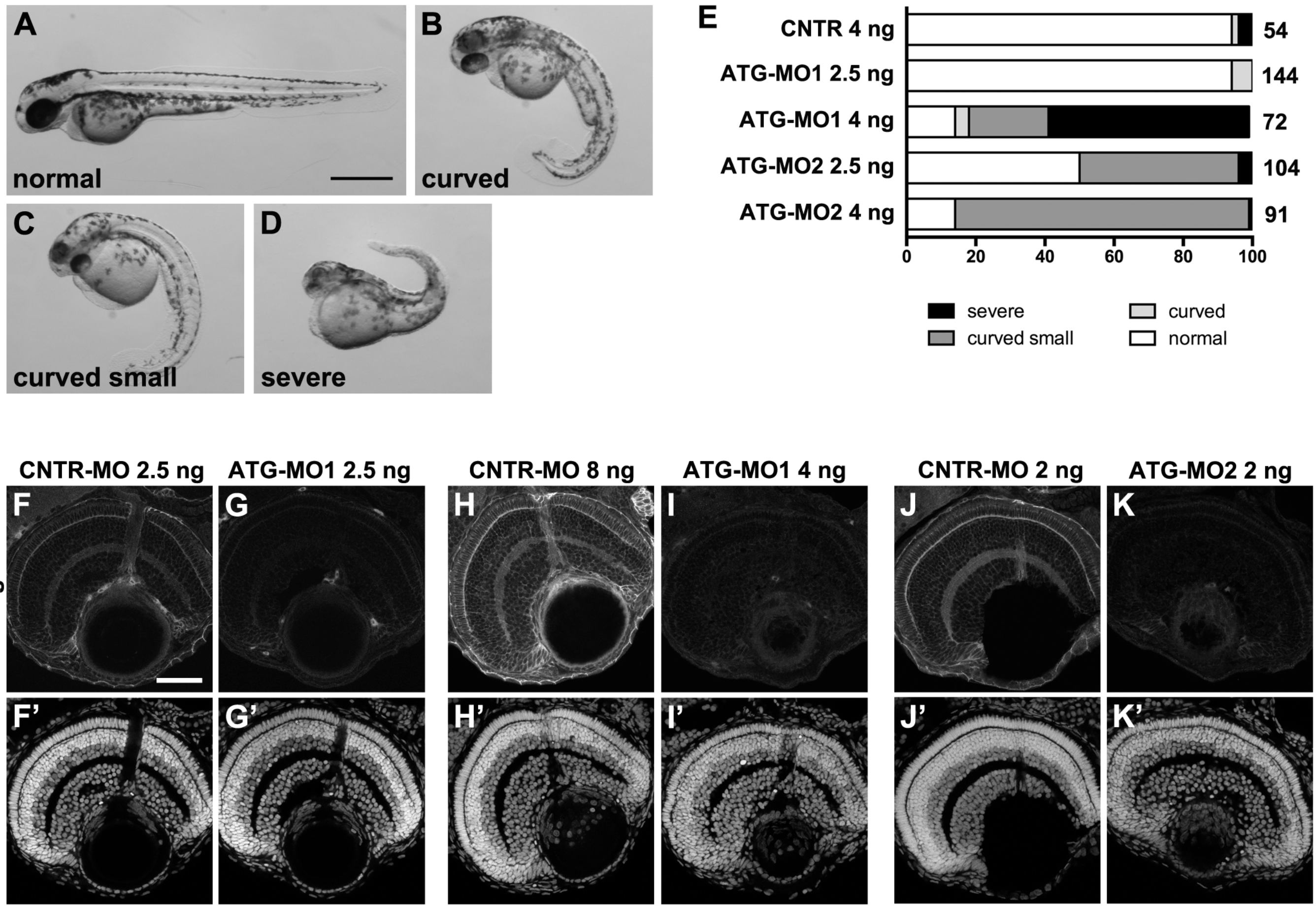

Figure S2. MO dose response and effect on Lgl2 protein expression in MO injected WT retinas. A-D. Variation of general phenotype at 2 dpf in morpholino-injected WT embryos. Scale bar, 0.5 mm. **E.** Frequency of phenotypes described in A-D at 2 dpf. Numbers at the end of bars denote total number of embryos analyzed. Graph shows the results of one representative experiment. **F-K'.** Immunostaining of transverse retinal sections at 3 dpf of MO injected WT animals. **F-G'.** 2.5 ng ATG-MO1 (G, G') injected per embryo with corresponding experimental control (F, F'). **H-I'.** 4 ng ATG-MO1 (I, I') injected per embryo with corresponding experimental control (H, H'). **J-K'.** 2 ng of ATG-MO2 injected per embryo (K, K') with corresponding experimental control (J, J'). Both MOs effectively reduce Lgl2 expression in the retina (compare F to G, H to I, and J to K). Scale bar, 50 μm.

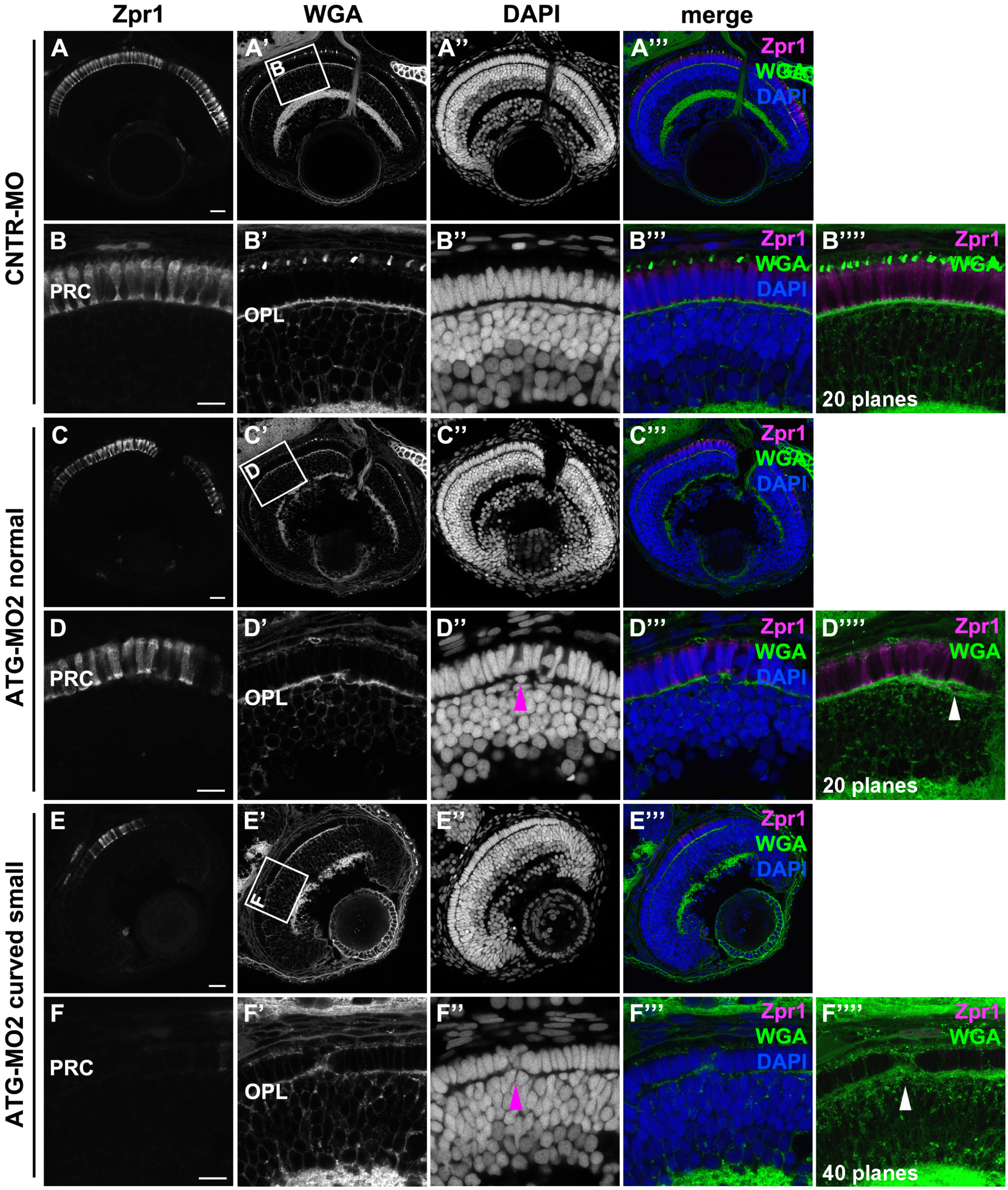

Figure S3. Knockdown of Lgl2 with ATG-MO2 in *pen/lgl2* background leads to a retinal phenotype. Immunostaining of transverse retinal sections at 3 dpf of ATG-MO2-injected *pen/lgl2* clutches **A-B''''.** Fish injected with control MO. **C-F''''.** Two examples of fish injected with ATG-MO2 with different severity of overall phenotype (see Fig. S2). Boxed regions in A', C' and E' show enlarged views of the distal retina. C-D'''. Retina of an ATG-MO2 injected fish that at 3 dpf displays a "normal" overall phenotype (Fig. S2A). Magenta arrowhead in D'' denotes a cluster of disorganized cells and white arrowhead in D'''' a breakage in the OPL clearly visible in a projection of 20 optical planes. **E-F''''.** Retina of an ATG-MO2 injected fish that at 3 dpf exhibits the "small curved" phenotype (Fig.

Biology Open • Supplementary information

S2C). Magenta arrowhead in F'' and white arrowhead in F'''' denote a cluster of disorganized cells. Occasionally, when looking at embryos with more severe overall morphological defects, we noticed a partial lack of Zpr1 signal in the PRC layer (E, F), suggesting an effect of ATG-MO2 on PRC differentiation. PRC, photoreceptor cell layer; OPL, outer plexiform layer. Scale bars: A, C, E, 20 µm; B, D, F, 10 µm.

Biology Open • Supplementary information
